# Supplementary material for: Elevational surveys of Sulawesi herpetofauna 2: Mount Katopasa on the Eastern Peninsula of Sulawesi island, Indonesia
Source: PeerJ. 2025 Sep 25;13:e20024. doi: 10.7717/peerj.20024 (PMC12476859; doi:10.7717/peerj.20024)

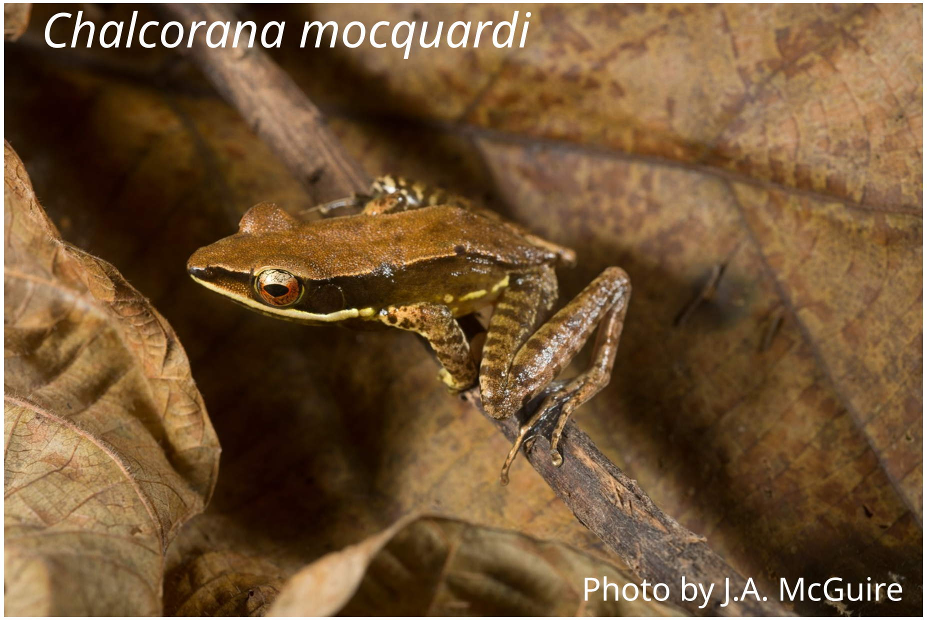
Chalcorana mocquardi
Photo by J.A. McGuire

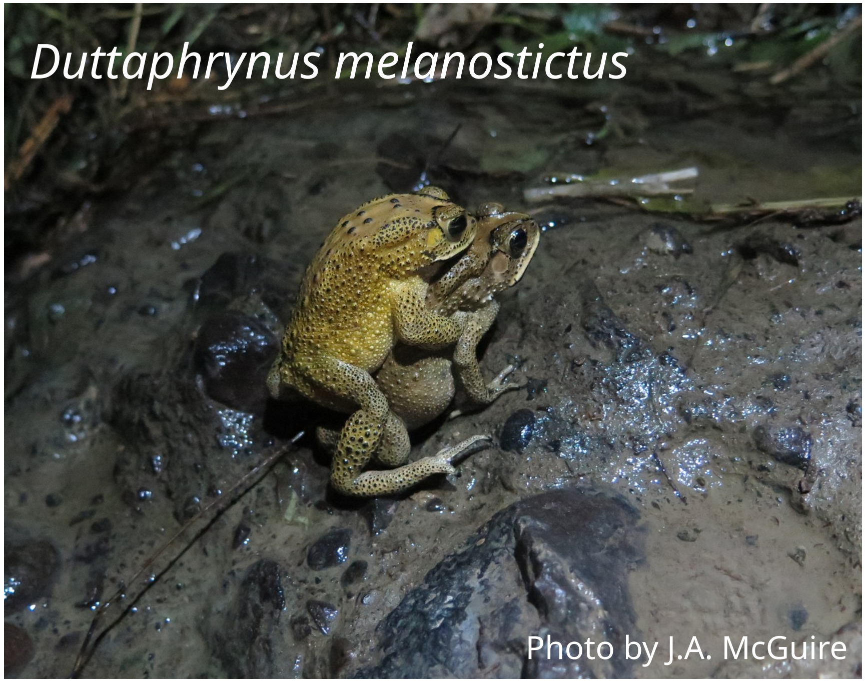
Duttaphrynus melanostictus
Photo by J.A. McGuire

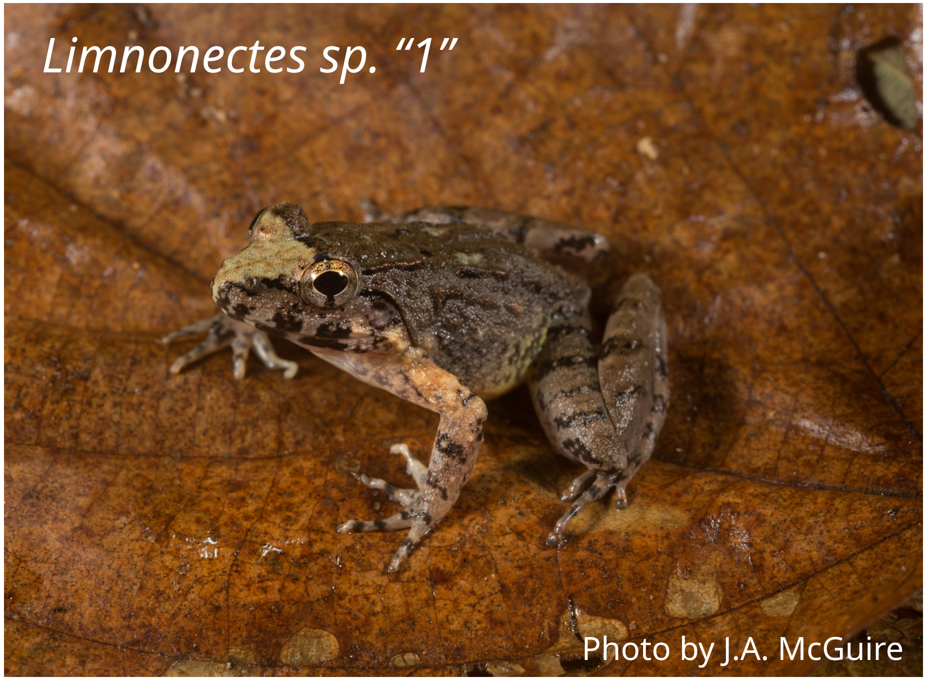
Limnonectes sp. "1"
Photo by J.A. McGuire

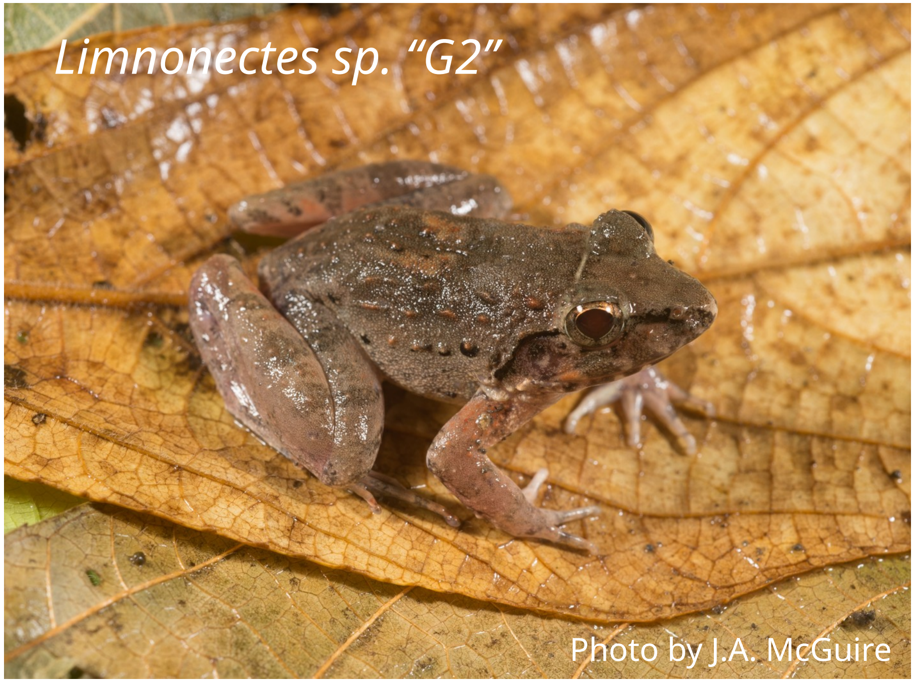
Limnonectes sp. "G2"
Photo by J.A. McGuire

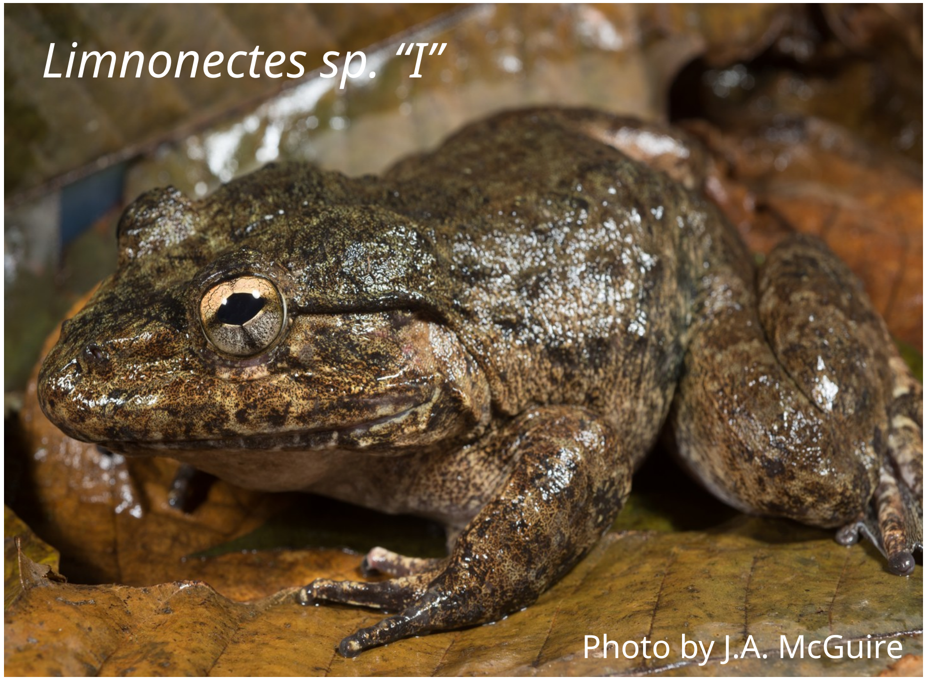
Limnonectes sp. "I"
Photo by J.A. McGuire

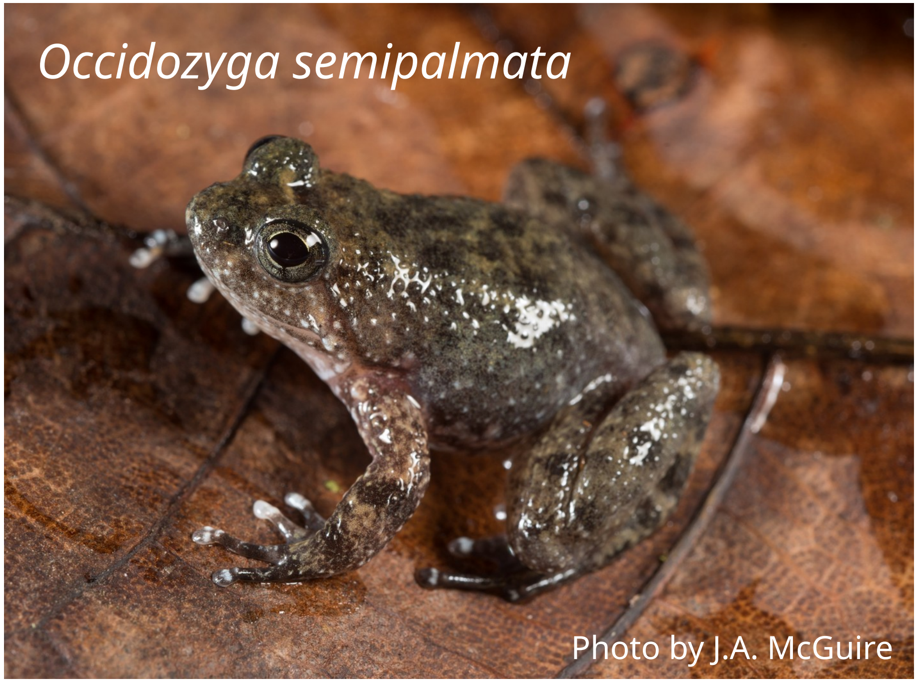
Occidozyga semipalmata
Photo by J.A. McGuire

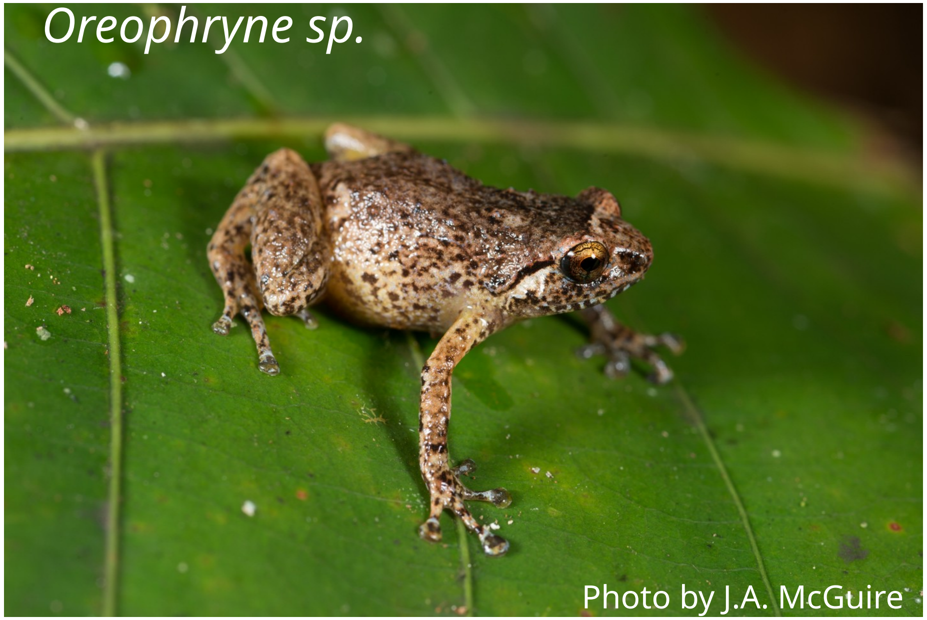
Oreophryne sp.
Photo by J.A. McGuire

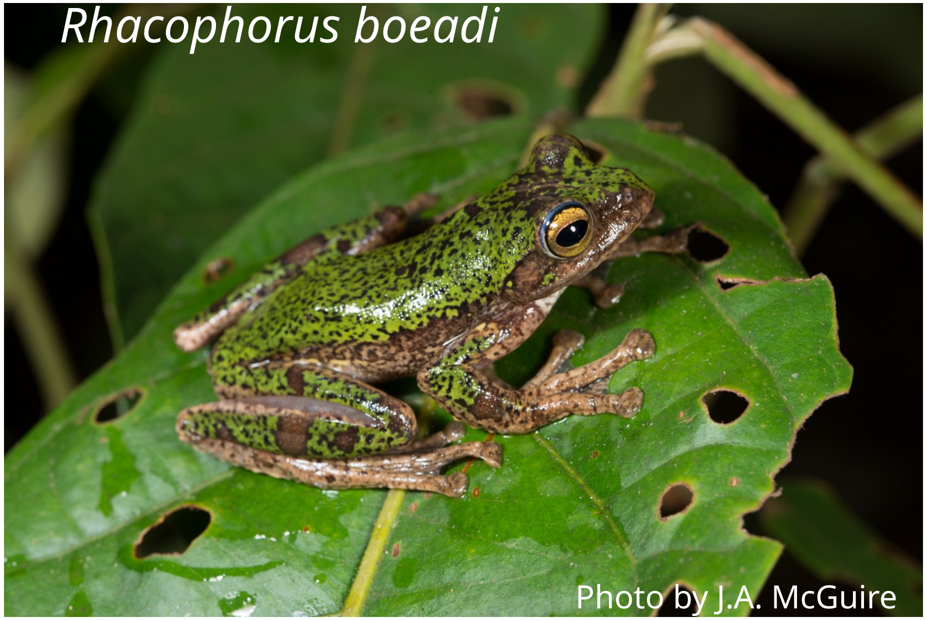
Rhacophorus boeadi
Photo by J.A. McGuire

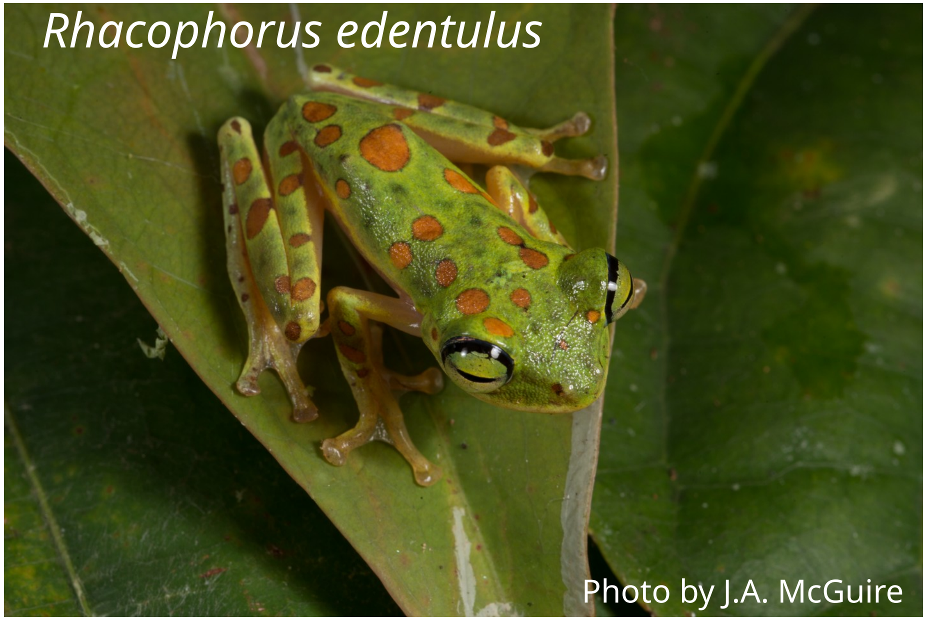
Rhacophorus edentulus
Photo by J.A. McGuire

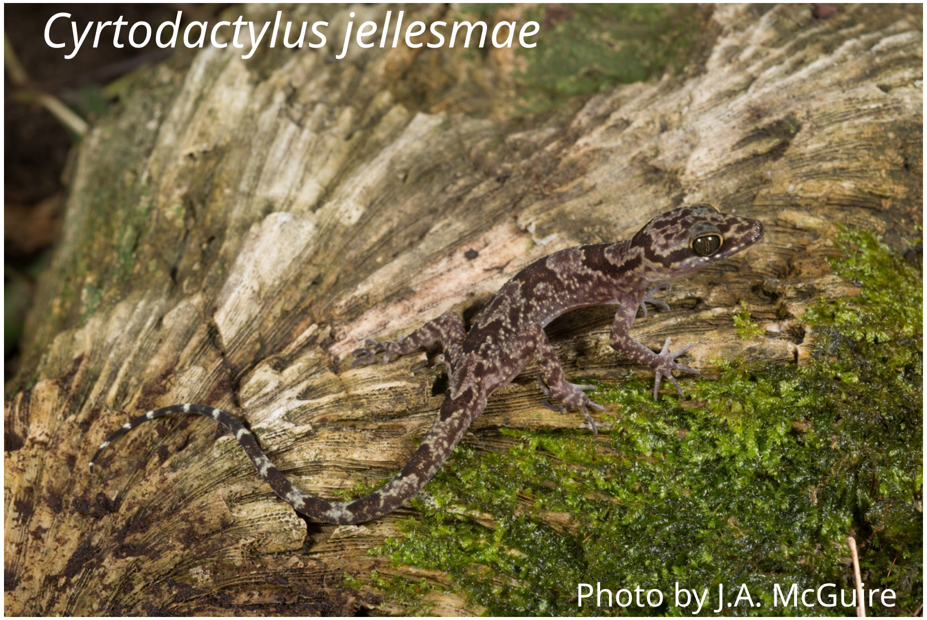
Cyrtodactylus jellesmae
Photo by J.A. McGuire

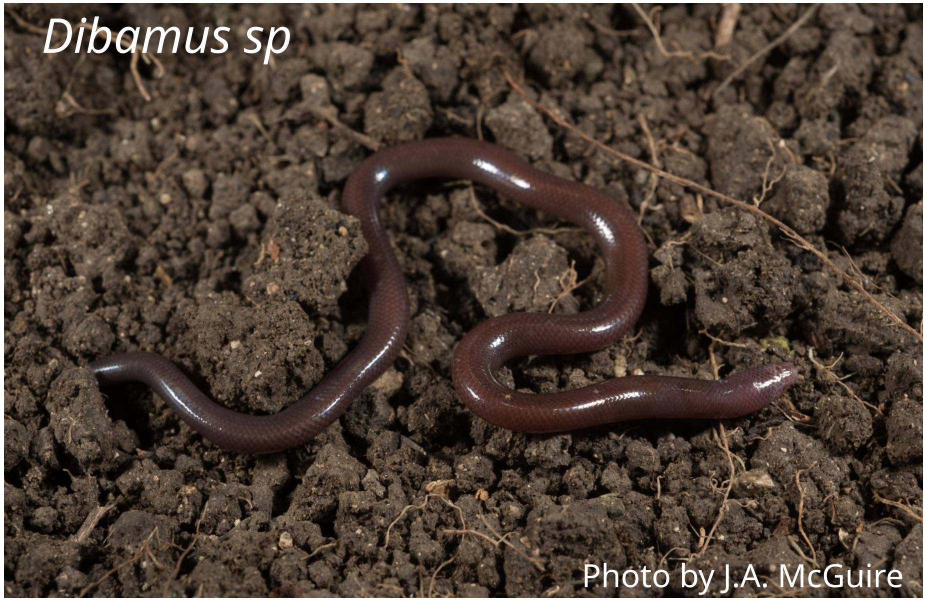
Dibamus sp
Photo by J.A. McGuire

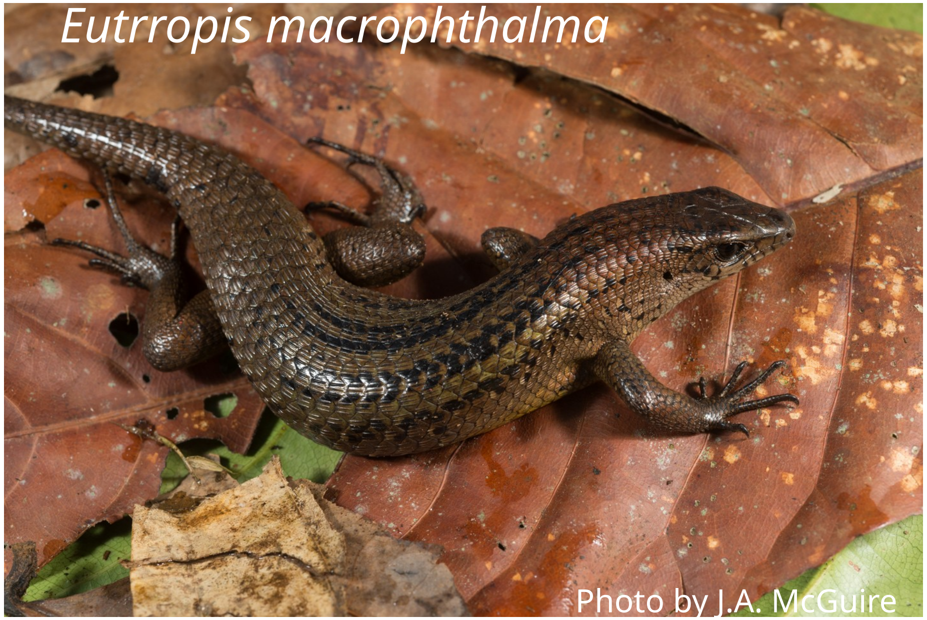
Eutrropis macrophthalma
Photo by J.A. McGuire

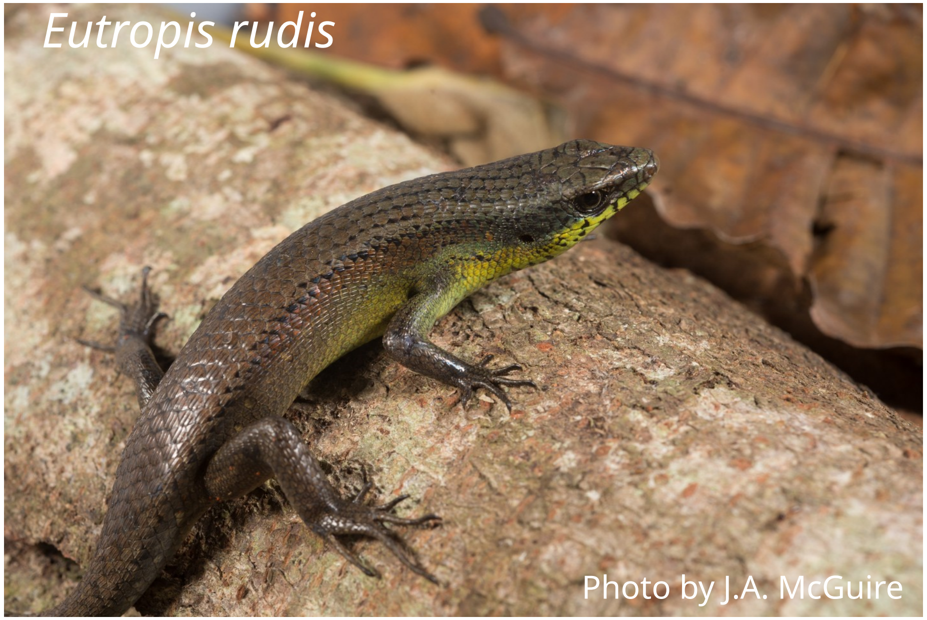

Eutropis rudis

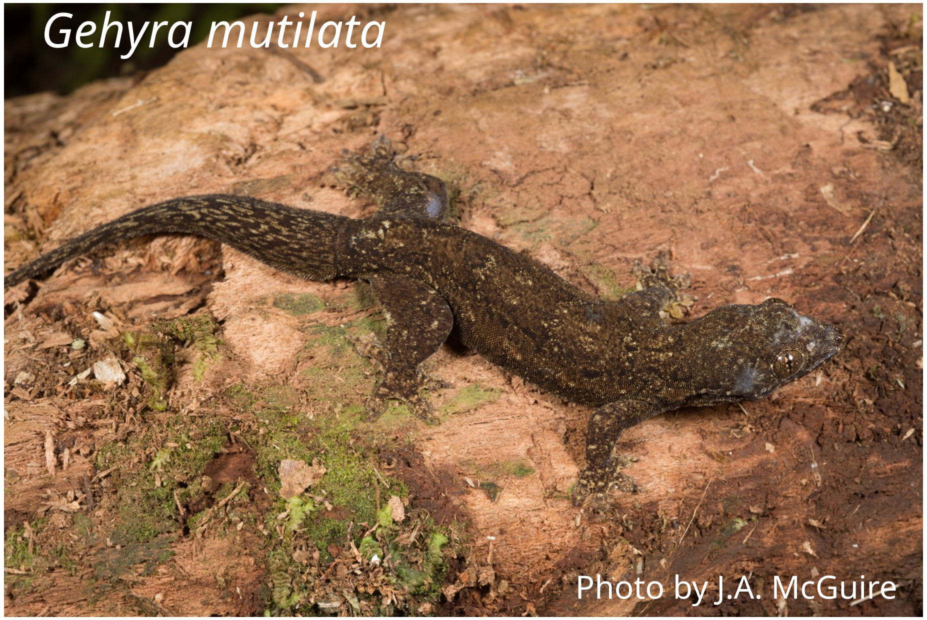

Gehyra mutilata

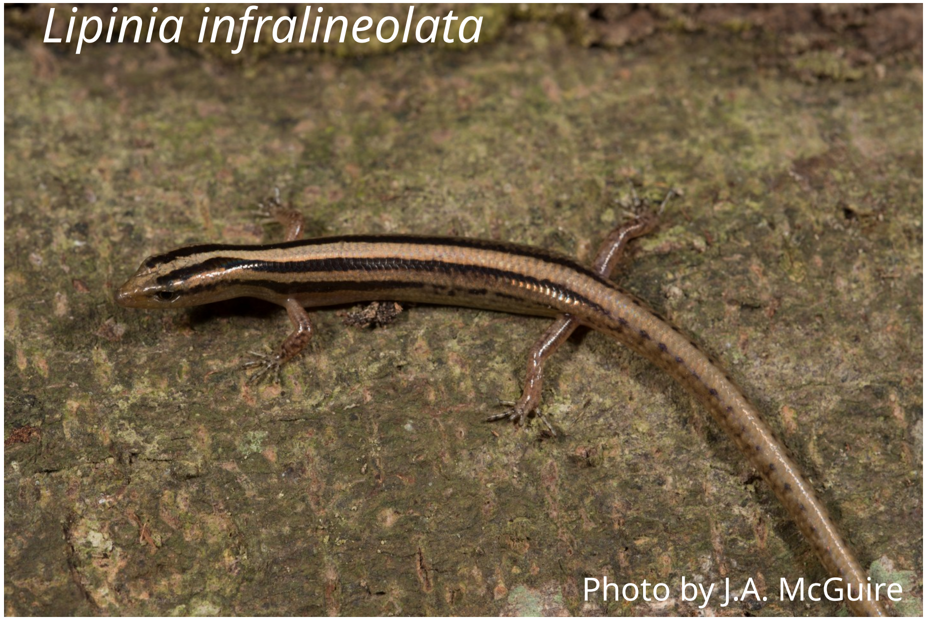

Lipinia infralineolata

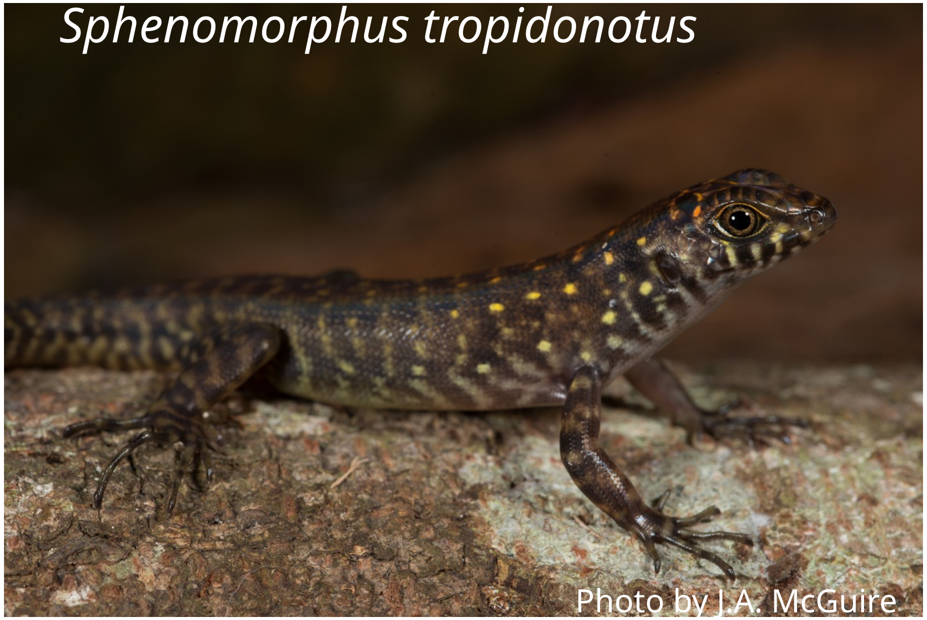

Sphenomorphus tropidonotus

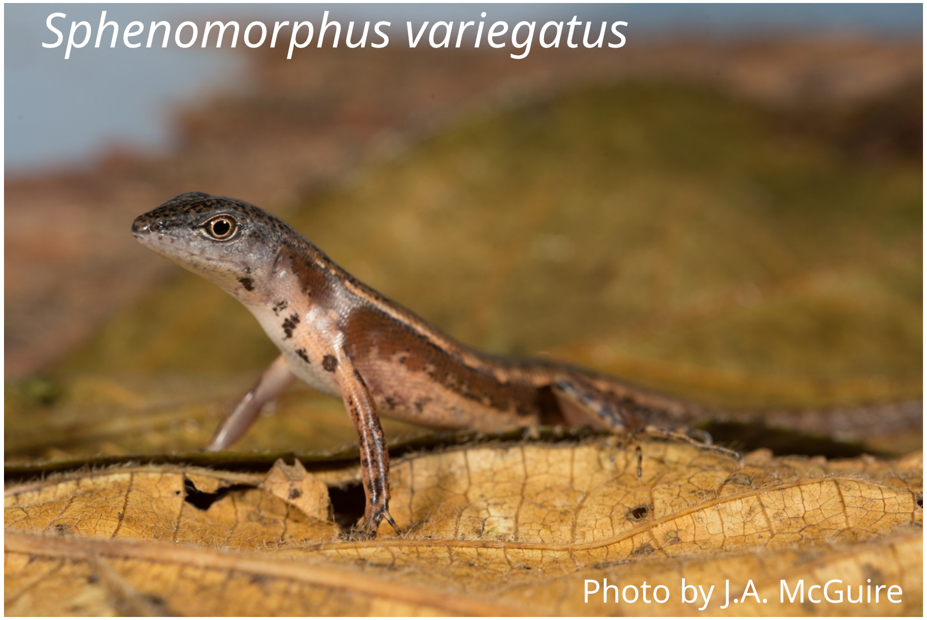
Sphenomorphus variegatus
Photo by J.A. McGuire

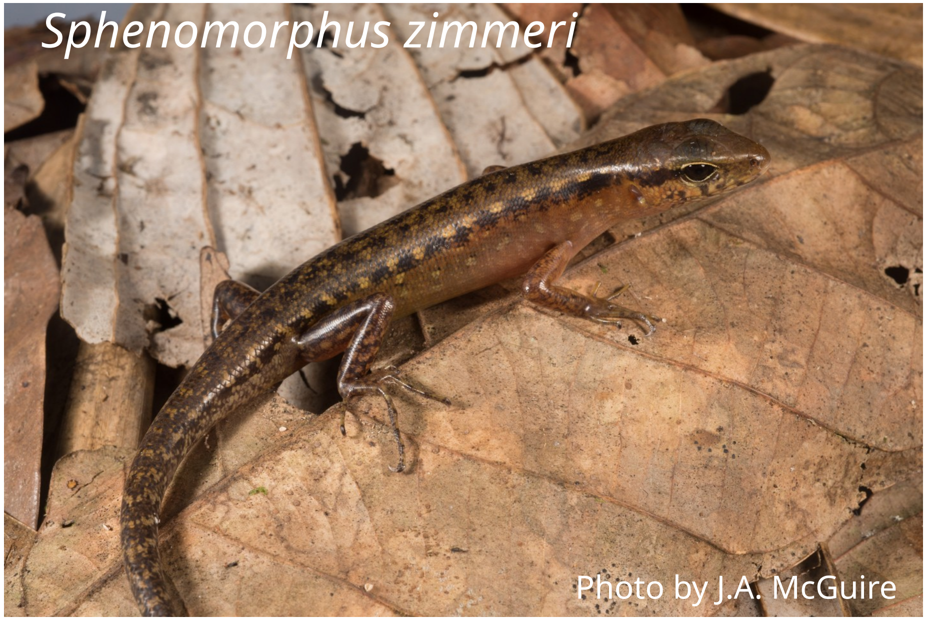
Sphenomorphus zimmeri
Photo by J.A. McGuire

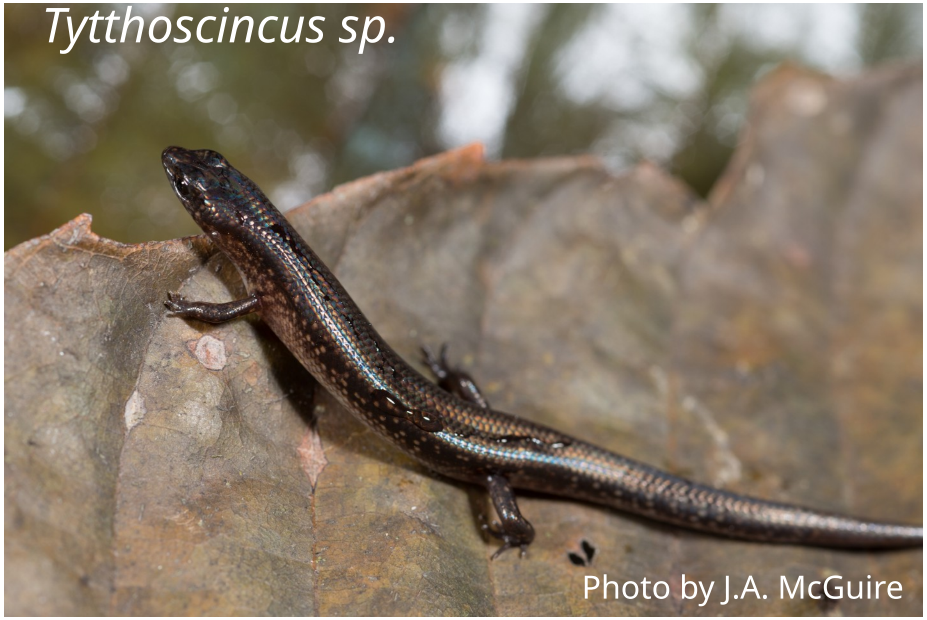
Tytthoscincus sp.
Photo by J.A. McGuire

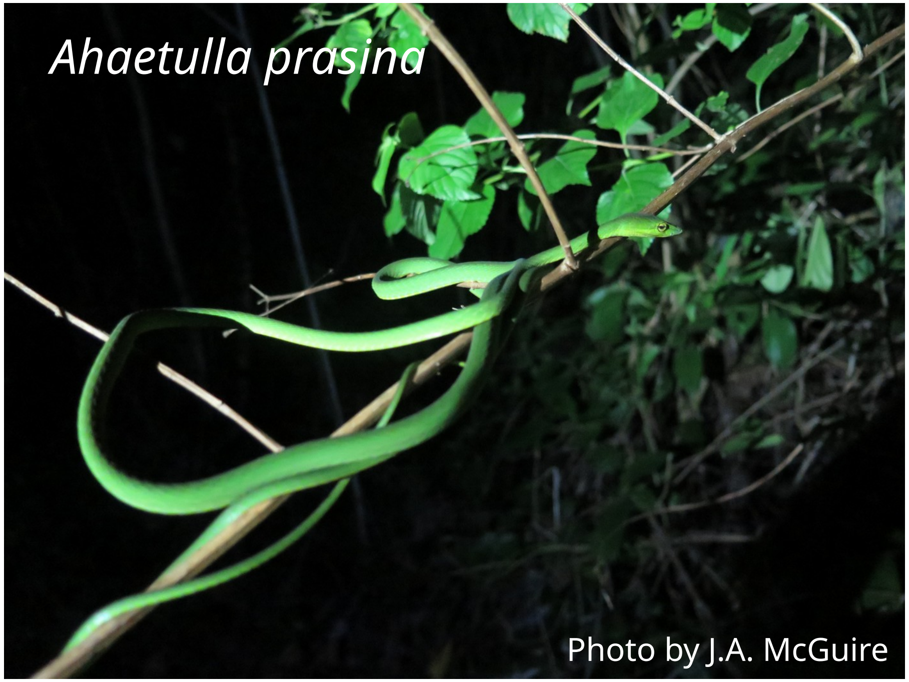
Ahaetulla prasina
Photo by J.A. McGuire

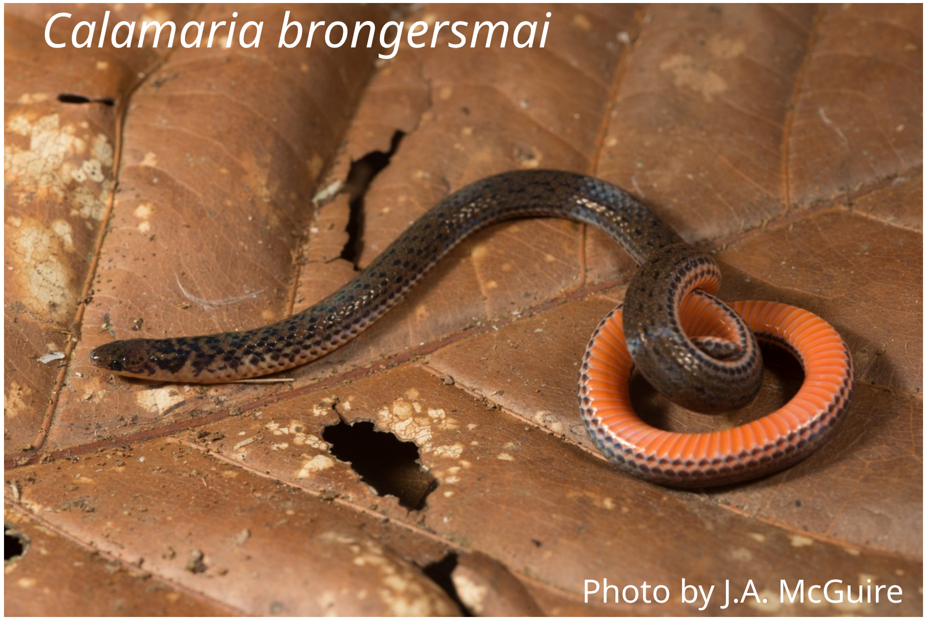
Calamaria brongersmai
Photo by J.A. McGuire

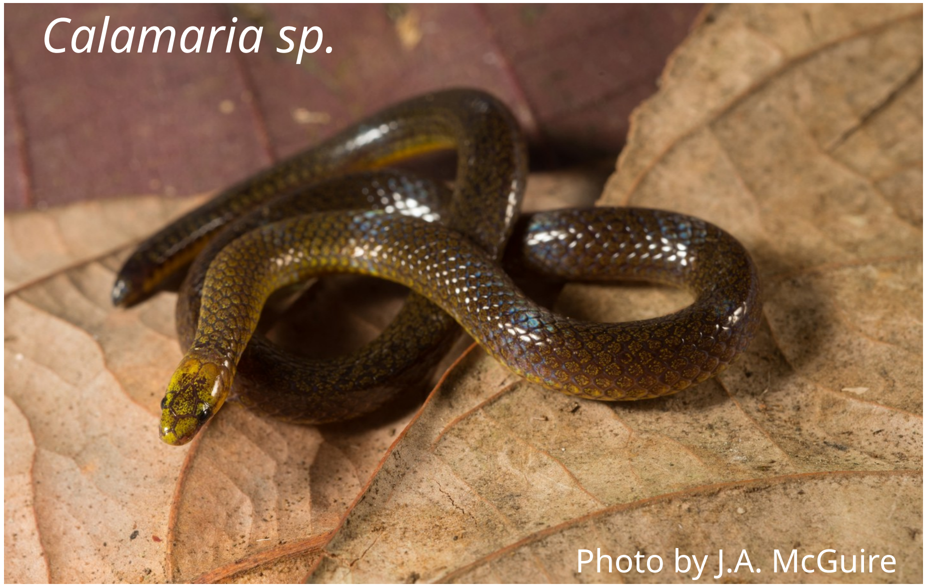
Calamaria sp.
Photo by J.A. McGuire

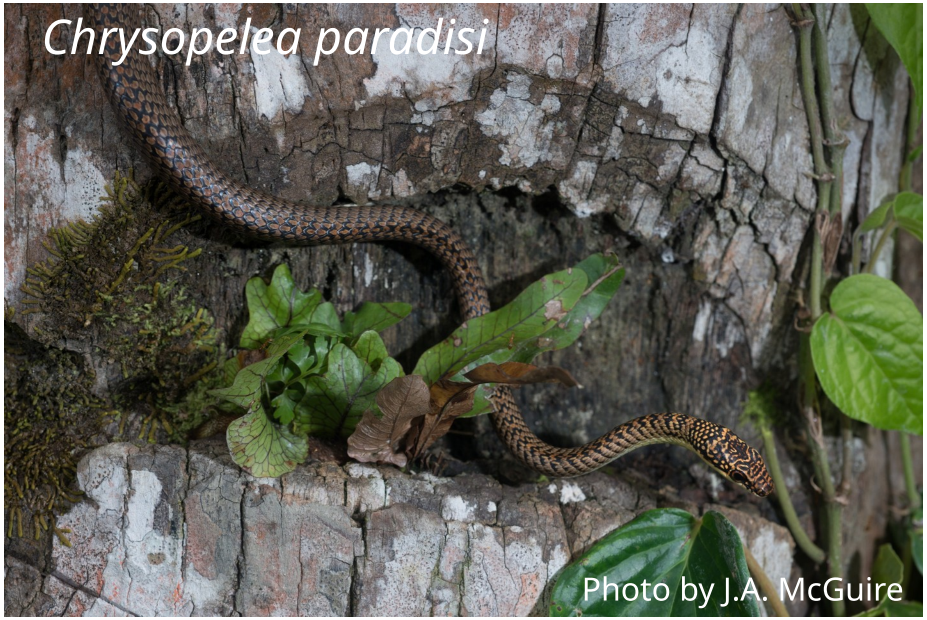
Chrysopelea paradisi
Photo by J.A. McGuire

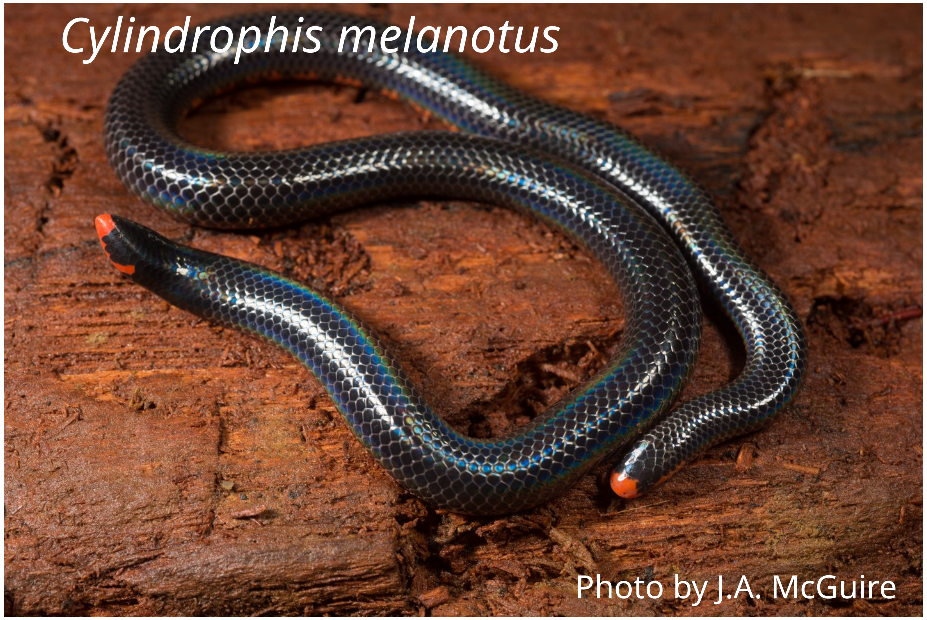
Cylindrophis melanotus
Photo by J.A. McGuire

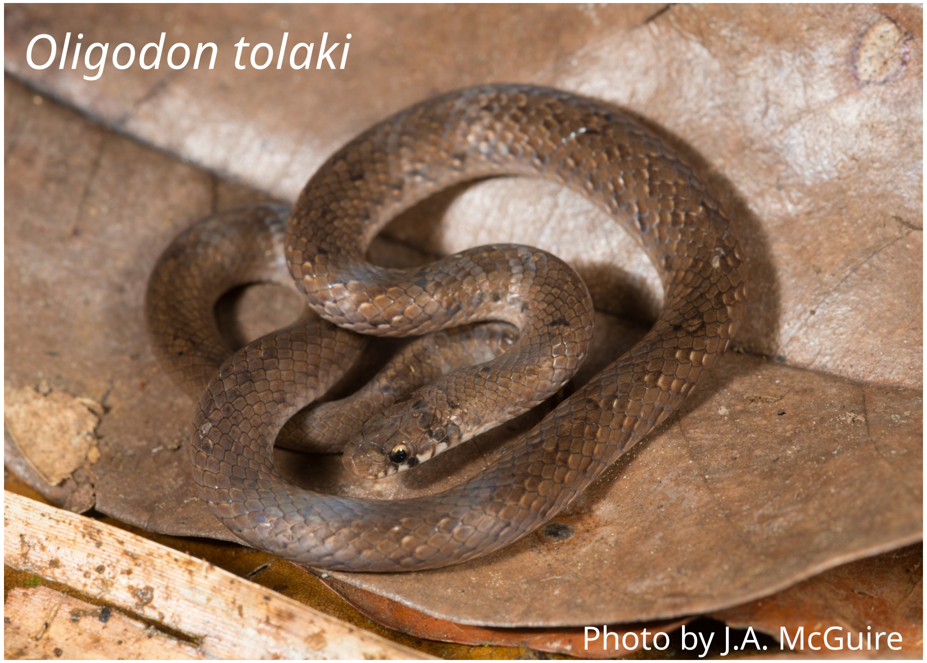
Oligodon tolaki
Photo by J.A. McGuire

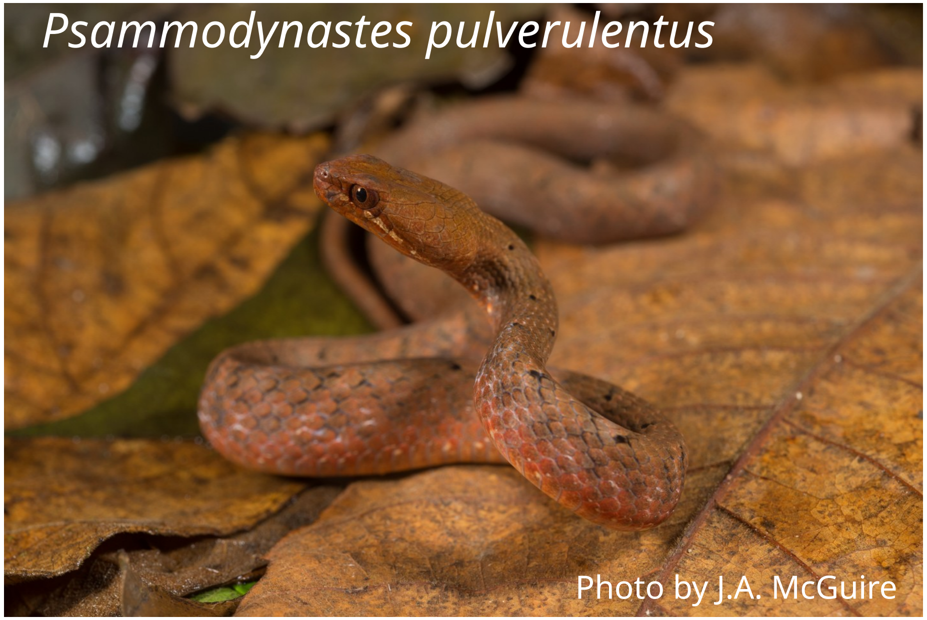
Psammodynastes pulverulentus
Photo by J.A. McGuire

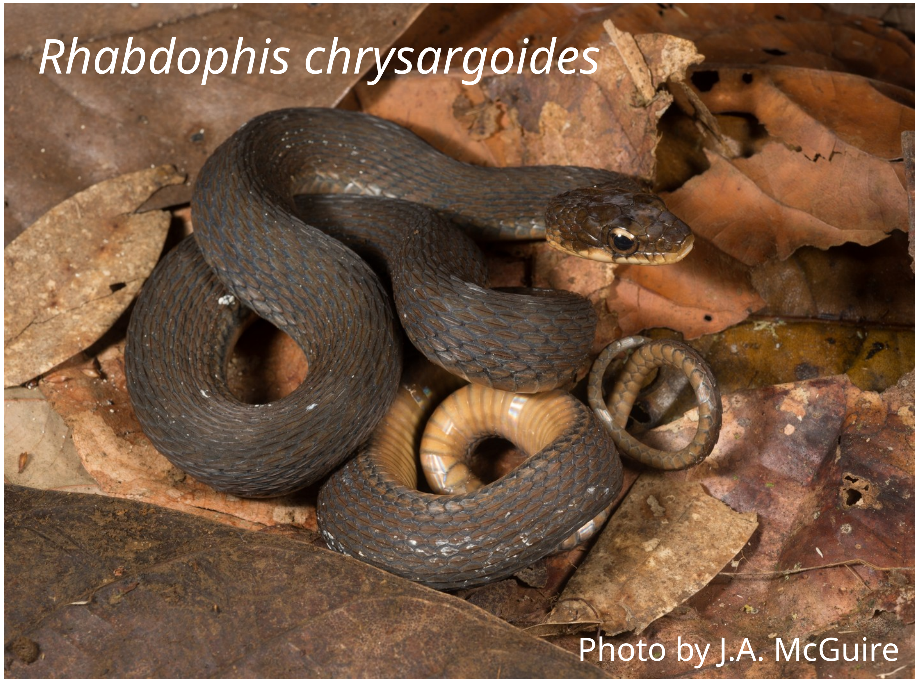
Rhabdophis chrysargoides
Photo by J.A. McGuire

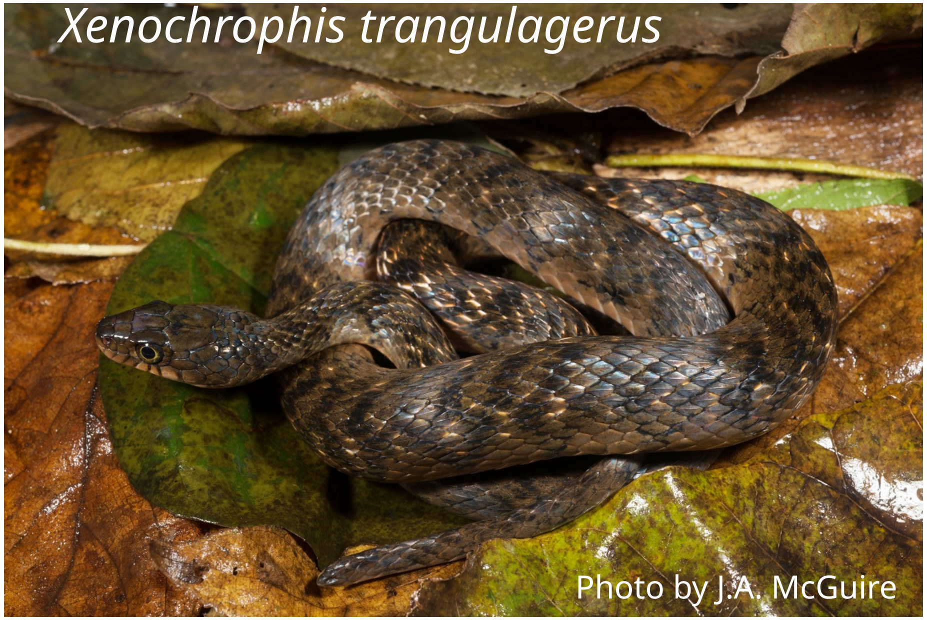
Xenochrophis trangulagerus
Photo by J.A. McGuire

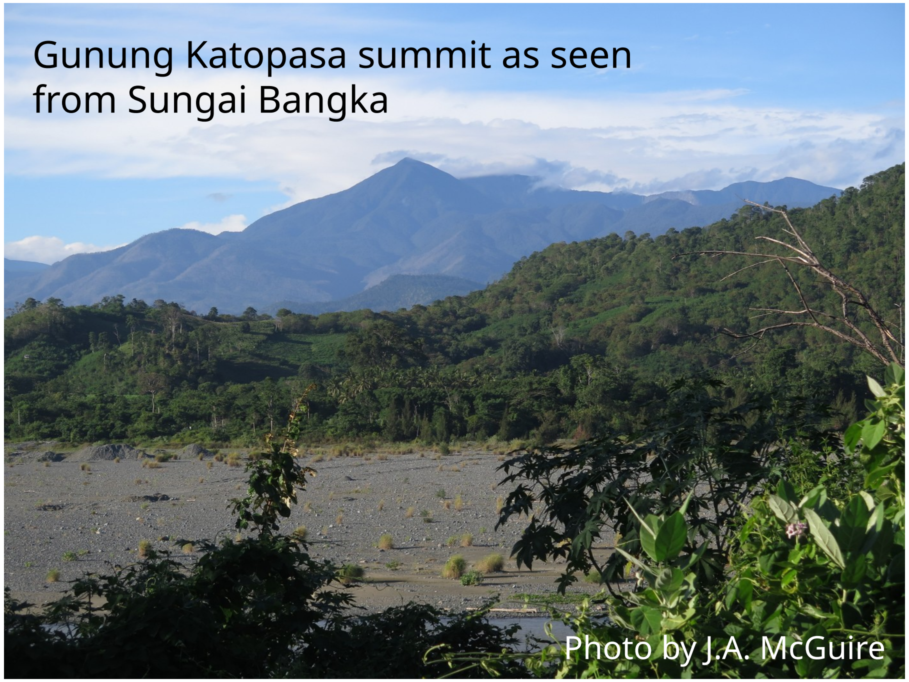

Gunung Katopasa summit as seen from Sungai Bangka

Photo by J.A. McGuire

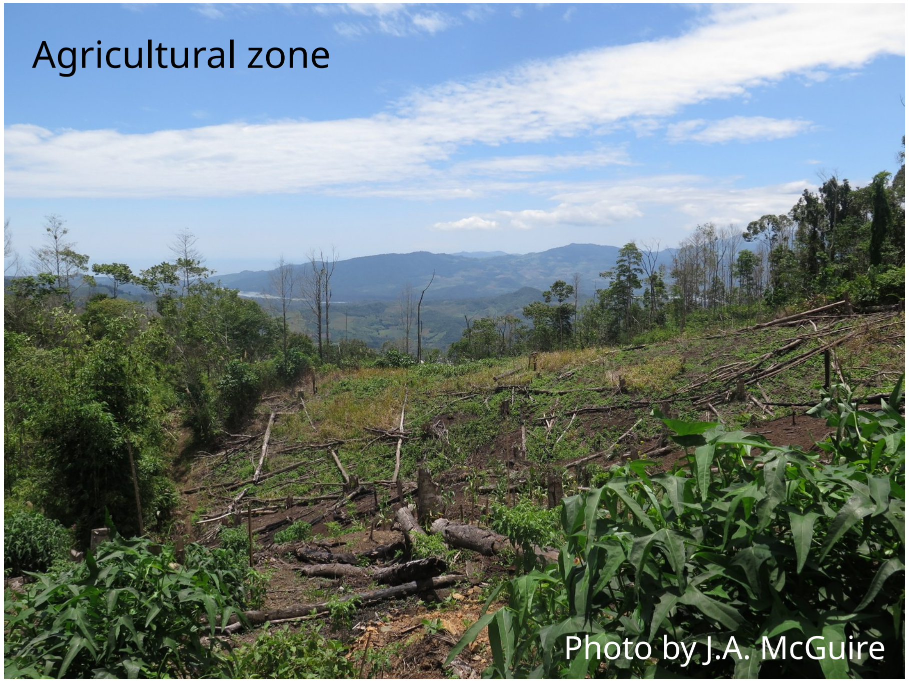

Agricultural zone

Photo by J.A. McGuire

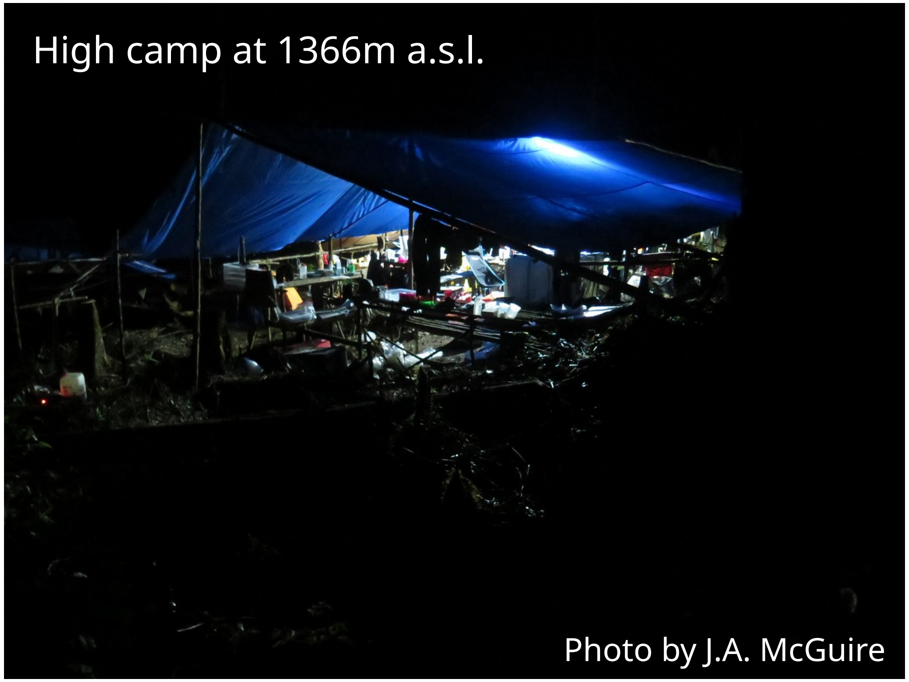

High camp at 1366m a.s.l.

Photo by J.A. McGuire

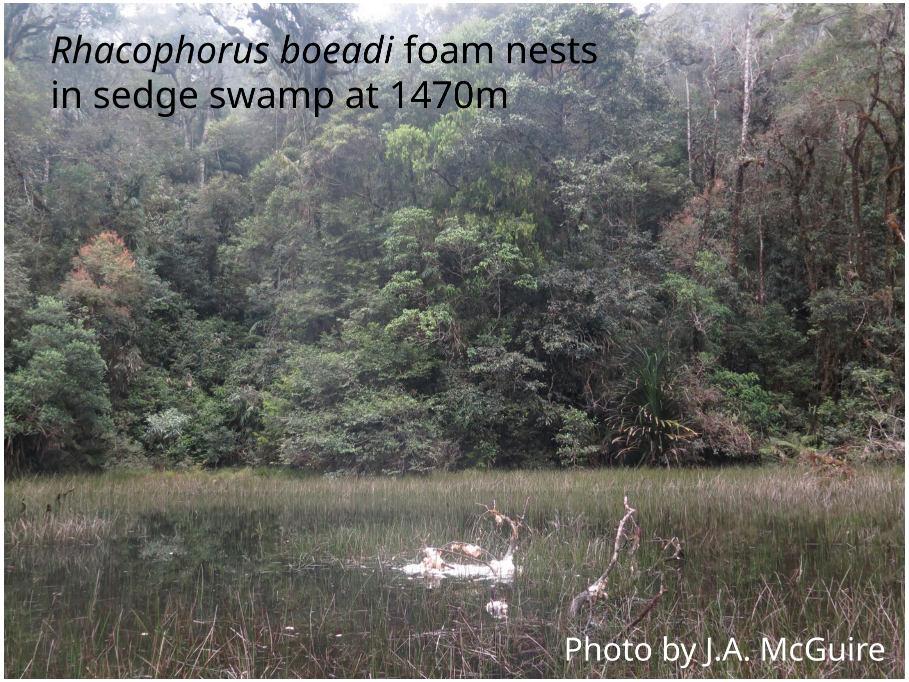

Rhacophorus boeadi foam nests in sedge swamp at 1470m

Photo by J.A. McGuire

Lowland stream environment.
Foreground: B. R. Karin

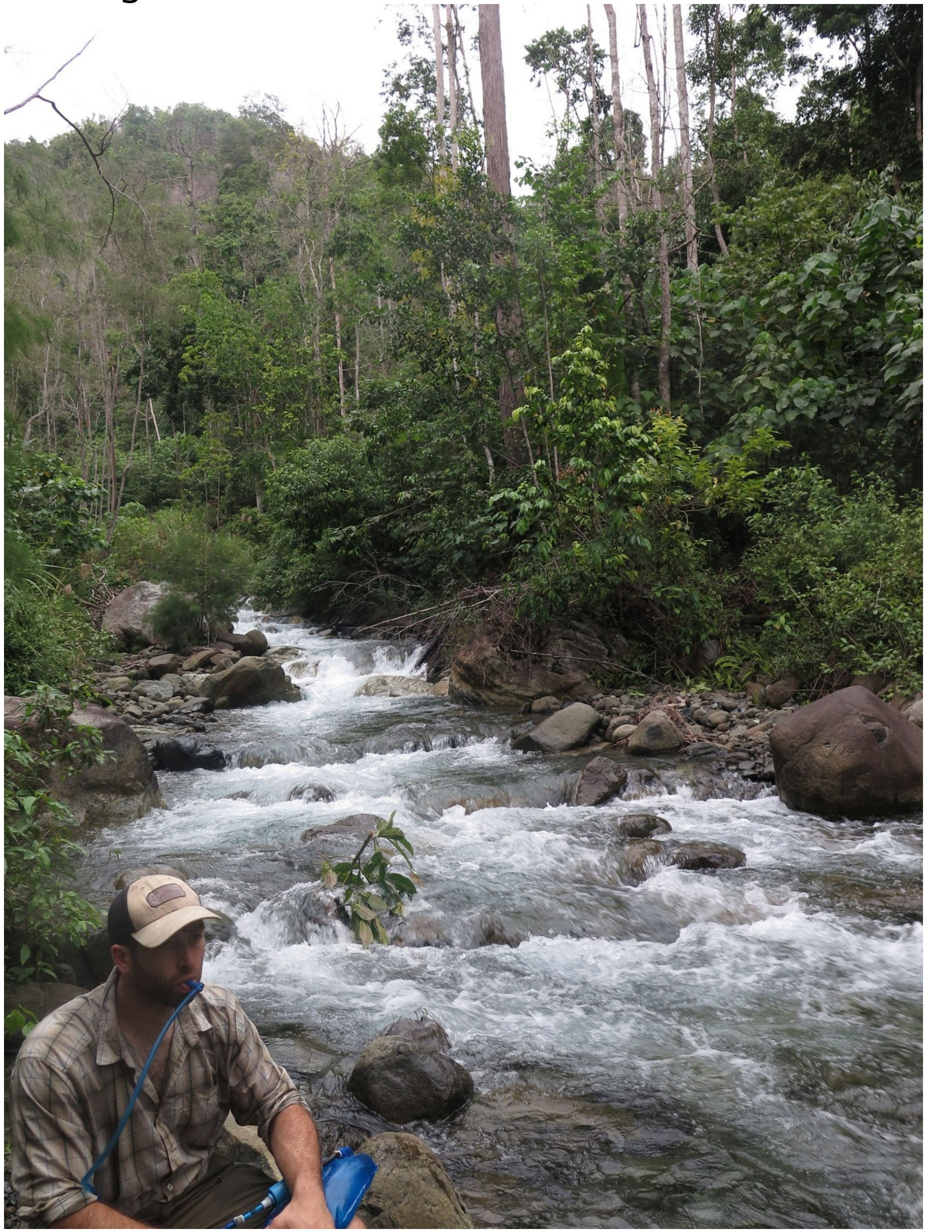

High-elevation mossy forest
Foreground: J. A. McGuire

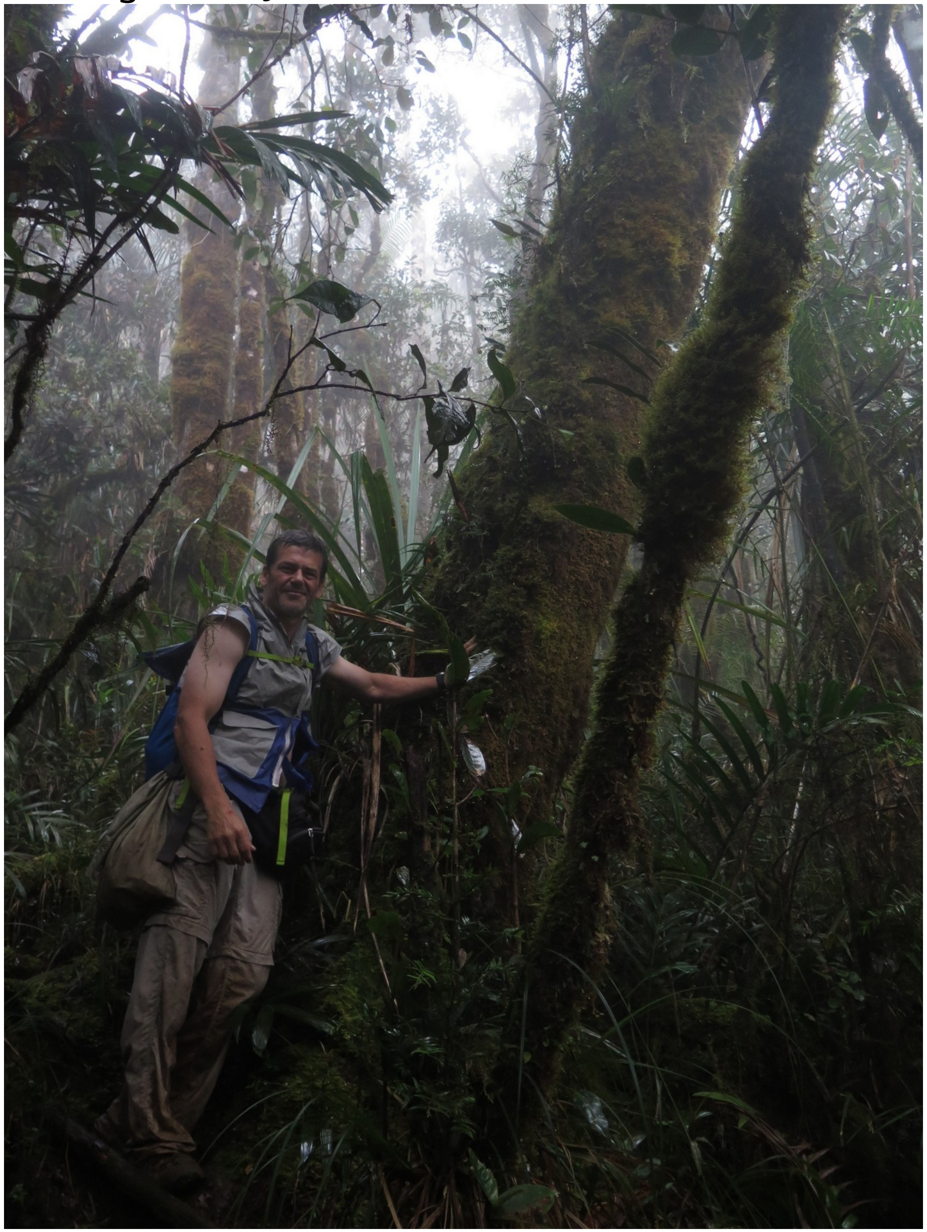

Supplement: Supplemental Information 1 [file peerj-13-20024-s001.pdf]
