# Supplementary material for: Elevational surveys of Sulawesi herpetofauna 2: Mount Katopasa on the Eastern Peninsula of Sulawesi island, Indonesia
Source: PeerJ. 2025 Sep 25;13:e20024. doi: 10.7717/peerj.20024 (PMC12476859; doi:10.7717/peerj.20024)
Supplement: Supplemental Information 3 [file peerj-13-20024-s003.docx]

**Abstrak (Bahasa Indonesia):**

Sejarah geologi pulau Sulawesi yang unik telah menghasilkan salah satu biota paling endemik di dunia, yang sebagian besar masih belum diketahui oleh sains. Biogeografi pulau ini dipengaruhi oleh bentuknya yang unik, yang merupakan pertemuan dari empat semenanjung, serta topografinya yang terdiri dari pegunungan, dan ekosistem dataran tinggi yang kurang dipahami oleh sains. Dalam artikel kedua dari serangkaian survei herpetologi di pegunungan Sulawesi yang dilakukan oleh tim peneliti internasional ini, kami menyajikan daftar lengkap spesies herpetofauna untuk Gunung Katopasa. Dalam tiga minggu survey di Gunung Katopasa (2825 m di atas permukaan laut) di bagian Semenanjung Timur Sulawesi, provinsi Sulawesi Tengah, kami menemukan 45 spesies reptil dan amfibi. Kami yakin bahwa setidaknya ada enam diantaranya yang merupakan spesies yang belum dideskripsikan. Hasil survey kami di Gunung Katopasa ini menunjukkan masih tingginya peluang menemukan keragaman spesies herpetofauna di Semenanjung Timur Sulawesi apabila penilaian mengenai kekayaan dan kelimpahan spesies di wilayah ini terus dilanjutkan.
